# Q&A: Understanding the composition of behavior

**DOI:** 10.1186/s12915-019-0663-3

**Published:** 2019-05-29

**Authors:** Sandeep Robert Datta

**Affiliations:** Harvard Medical School Department of Neurobiology, WAB 336, 200 Longwood Avenue, Boston, MA 02115 USA

## Abstract

Understanding the brain requires understanding behavior. New machine vision and learning techniques are poised to revolutionize our ability to analyze behaviors exhibited by animals in the laboratory. Here we describe one such method, Motion Sequencing (MoSeq), which combines three-dimensional (3D) imaging with unsupervised machine learning techniques to identify the syllables and grammar that comprise mouse body language. This Q&A situates MoSeq within the array of novel methods currently being developed for behavioral analysis, enumerates its relative strengths and weaknesses, and describes its future trajectory.

## Why do we need new methods to analyze the behavior of animals in the lab?

We have long had powerful tools to condition animals and to measure their trained behavioral responses. Recent advances in computing have allowed researchers to graft onto this basic framework all sorts of bells and whistles—like graphics engines that build complete virtual worlds for rodents to explore, or systems that automatically train rodents for weeks at a time in complex timing or discrimination tasks—that are enabling ever deeper insight into brain function. What has been missing are equally powerful tools for understanding the behavior of animals that are unrestrained (both in the physical sense and in the broader sense of not having been overtrained to express a particular action in response to a cue). In the wild, animals generate spontaneous and self-directed actions to explore and interact with the world, and arguably a main function of the brain is to support these sorts of ethologically relevant and naturalistic patterns of action. However, it remains a significant technical challenge both to estimate poses and movement parameters of animals during unrestrained behavior, and to characterize how these poses and movements are meaningfully organized over time.

## How is this problem being addressed with modern tools?

There are many new approaches being developed to measure and analyze spontaneous, unrestrained behavior. Let’s talk first about the input side, about how we can measure things. If you are willing to mount devices on your rats or mice, you can now get movement data from accelerometers, which are often integrated into headstages used for electrophysiology. More complicated devices, combining mirrors and miniaturized head-mounted cameras, enable ongoing measurements of pupil dilation and whisking [1]. But perhaps the most broadly impactful set of new methods derives from improvements in computational algorithms to perform point tracking in videos of behavior. Over the last few years several custom and generalizable pipelines have been described (e.g., JABAA, Optimouse, LocoMouse, LEAP, and DeepLabCut to name a few) that allow users to identify keypoints in videos (like the centroid of a mouse, or the location of a paw), and to track the movement of those keypoints across video frames automatically and with high accuracy [2–6]. These approaches are transformative for our ability to capture parametric behavioral information from video, particularly given the impressive deep learning-based performance of frameworks like LEAP and DeepLabCut [5, 6]. In parallel with the advent of better tracking code, improvements have also been made in depth sensing technology, thereby enabling facile 3D imaging of rodents as they behave in the lab [7].

## Wait, I thought you said behavior wasn’t a solved problem—it seems pretty solved to me! So, we are done now, right?

Not quite—there is the issue of actually understanding all these new datastreams. Which is something that none of these tools, on their own, are designed to do.

## What do you mean by “understanding” these data?

“Understanding” behavior means different things to different people, of course, and is very much dependent upon the goals of the experimenter. If you are tracking paws during reaches for pellets, for example, then what you care about are the paw trajectories themselves; this means that from a behavior perspective, point tracking alone gets you very close to where you need to be. Problems like tracking whisker movements (which allow rodents to probe their environments through touch) or pupil dilation (which reflects arousal) fall into this category.

However, there are many problems for which behavioral data aren’t so transparently informative on their own, and instead have to be organized and parsed to gain insight. Labeling behaviors is one such problem; in this case, the experimental challenge is to associate patterns in behavioral data (e.g., movement parameters extracted from a mouse behavioral video) with labels that humans can directly or indirectly interpret (e.g., running, rearing, eating, sleeping). The most straightforward way to address this problem is through supervised machine learning, where human-labeled video data are used to train classifiers that automatically identify the behavioral state being expressed at each point in time. However, humans are notoriously bad animal psychologists, and human supervision comes with inevitable biases about what constitutes a meaningful unit of behavior—which nearly always corresponds to behaviors for which humans already have natural language descriptors.

To elide this limitation, many labs have begun applying recently developed unsupervised machine learning techniques, which parse behavior into units of action based upon underlying statistical structure without relying upon explicit human-supplied labels. These attempts to identify regularities in behavioral data find their origin in the work of the ethologists, who posited that behavior is modular—that is, made up of repeated and stereotyped behavioral motifs—and who predicted that the brain would flexibly compose behavior by stringing together behavioral modules or motifs into different sequences. As was true among the ethologists 50 years ago, there are vigorous discussions underway about how best to identify latent structure in behavioral data. My lab’s participation in this conversation started several years ago when we developed a technique called Motion Sequencing, or MoSeq for short [7].

## What is MoSeq?

MoSeq is a technique that uses 3D machine vision and unsupervised machine learning to automatically discover the underlying modular structure of mouse behavior in the lab [7–10]. It has two distinct and separable components. The first is about measuring behavior—the current versions of MoSeq use depth cameras (which can be placed above any behavioral arena) to measure how the 3D poses expressed by a mouse change over time. These data constrain what MoSeq thinks a module of behavior is, because the data being modeled arise from a single 3D camera looking down at an arena from a single axis of view, which captures only the top two-thirds of the mouse’s body. As a consequence, these data represent overall 3D body movements, but do not directly capture many aspects of the mouse’s behavior.

**Fig. 1.**
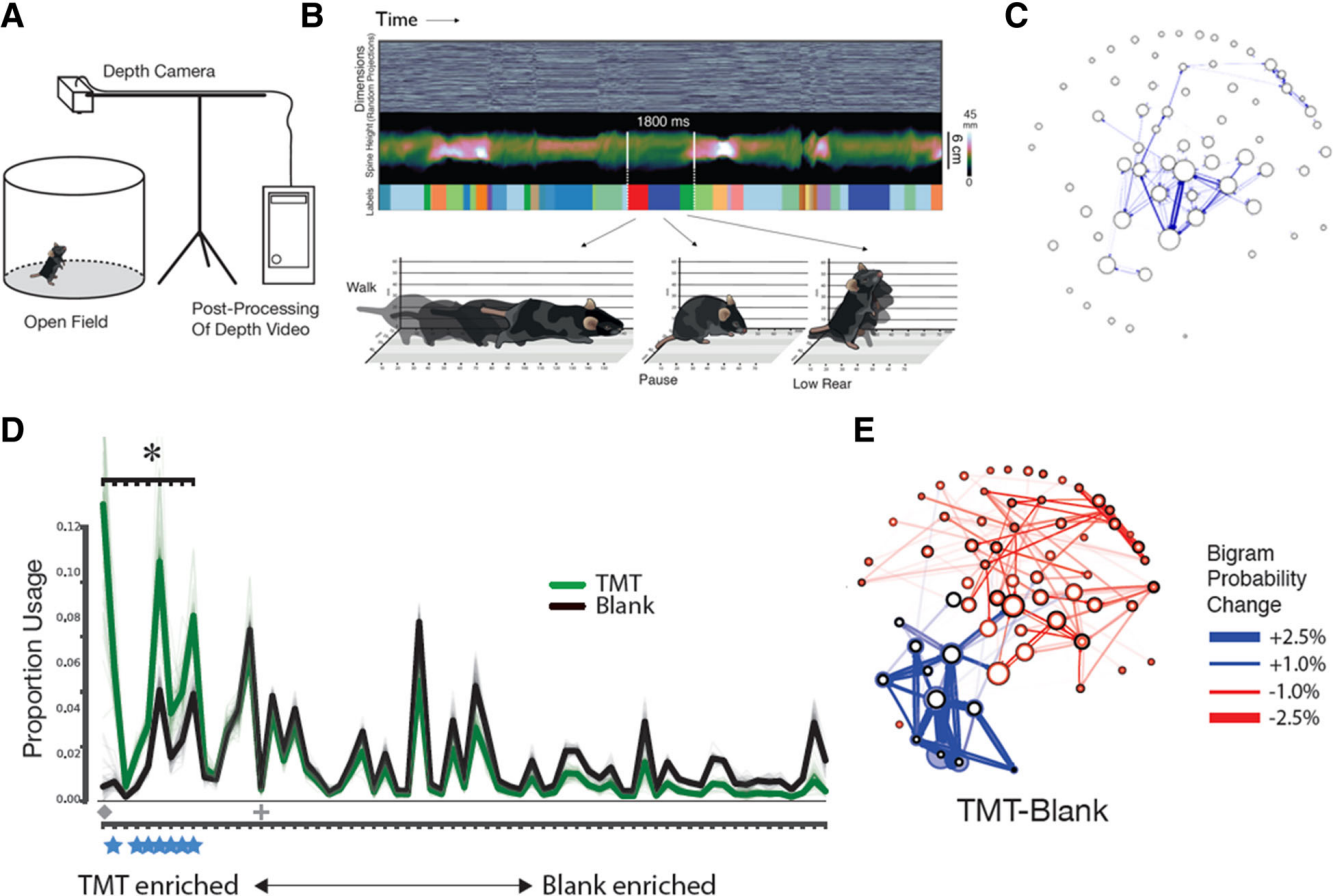
**a** 3D imaging of mouse pose dynamics. MoSeq uses depth cameras to image the 3D pose dynamics of mice, which are then used to identify behavioral syllables and grammar. **b** Plotting the imaged 3D behavioral data over time (compressed here using the random projection technique, *top row*, *grey*) reveals that behavior self-organizes into blocks (apparent as vertical striations in the imaging data). Plotting the mouse’s height at each point along its spine (*middle row*) similarly reveals the block-like dynamics of the mouse’s behaviors during the experiment. Based upon the intrinsic structure present in the data, MoSeq uses a probabilistic modeling approach to identify the complete set of behavioral syllables expressed within the experiment, and then takes advantage of this information to label each frame of 3D video (*bottom row*, indicated as *colored blocks*). Each discovered behavioral syllable is a brief, reused, and stereotyped motif of action (*bottom*); in a typical 30-min experiment in a featureless bucket approximately 40 such syllables are identified that encapsulate 95% of the mouse’s behavior. **c** Behavioral state maps generated by MoSeq depicting behavioral syllables (nodes, diameter is proportional to syllable usage) and transitions (edges, thickness is proportional to transition likelihood) encapsulate all behavior expressed within a given experiment captured by the camera and can be used to predict future behavior from present actions. **d** Exposing mice to stimuli (or manipulating genes or neural circuits, not shown) causes changes in the overall usage of individual syllables during an experiment. Here, mice were exposed to the fox odorant TMT, which causes fear-like behaviors in the mouse, including avoidance of the odor source. Exposure induces a wholesale behavioral state change in the mouse, which can be captured as differences in syllable expression (*asterisk* indicates syllables that pass a statistical test for difference with air-exposed mice). **e** Plotting out behavioral state maps for control and TMT conditions (as in **c**), and then subtracting these state maps identifies new behavioral trajectories through syllable space that are induced by exposure to the stimulus. Upregulated temporal connections between syllables are shown in *blue*, while downregulated connections are shown in *red*. The new behaviors induced in mice by TMT—including freezing and avoidance—are encoded by trajectories through the *blue* part of this state space. Figure (parts **b-e**) adapted from [7] Neuron 88(6), Alexander B. Wiltschko, Matthew J. Johnson, Giuliano Iurilli, Ralph E. Peterson, Jesse M. Katon, Stan L. Pashkovski, Victoria E. Abraira, Ryan P. Adams, Sandeep Robert Datta, Mapping sub-second structure in mouse behavior, 1121–1135., Copyright 2015, reprinted with permission from Elsevier

The second component is about understanding behavior. MoSeq is based upon a generative computational model whose structure reflects the ethological hypothesis that behavior is built out of a set of identifiable behavioral modules (which are described mathematically as autoregressive processes through pose space) that tumble one after the other somewhat predictably in time (a process that is described through a semi- or sticky-hidden Markov model). Based upon statistical regularities in the pose dynamics expressed by mice in an experiment, as well as the sequence over time in which those pose dynamics are expressed, MoSeq uses probabilistic fitting procedures to automatically identify the behavioral modules and sequences that optimally explain the observed pattern of behavior within any given experimental dataset (Fig. 1). MoSeq is also flexible, insofar as the number and contents of the modules are not predefined—they are learned from the data through unsupervised machine learning methods.

The model underlying MoSeq asserts that mice can only do one thing at a time, which means that if there is a “chewing gum” module and a “walking” module, and mice chew gum and walk at the same time, MoSeq will identify a “walking and chewing gum” syllable that is different from its components. In addition, from the perspective of the model mice are always doing *something*: as a consequence, each imaged frame is obligately assigned to a single identified module. We refer to the modules identified by MoSeq as behavioral syllables, and the statistics that govern their interconnection over time as behavioral grammar.

## What do behavioral syllables and grammar look like?

Syllables are brief, 3D motifs of behavior; typically these are actions like a specific type of rear, or a head-bob to the left, or a distinct form of pausing behavior (Fig. 1b). Each instance of a MoSeq-identified syllable is stereotyped, but not perfectly, as MoSeq assigns each syllable its own spatiotemporal distribution to account for intra-syllable variation. While the average duration of a behavioral syllable is about 300 ms, this can vary quite a bit. The temporal relationships between syllables can be visualized using a state map, where the nodes are syllables and the edges are observed transitions between syllables (Fig. 1c). One thing that immediately pops out from generating such state maps is that mouse behavior is highly structured—there appear to be a set of rules that govern how different behavioral syllables can be put together over time to create continuous patterns of action.

## How does MoSeq discover how many parts of behavior there should be?

MoSeq is a generative model, which means that once MoSeq identifies a candidate set of syllables and grammar from a given dataset, a synthetic “movie” can be played in which MoSeq predicts what a mouse’s behavior should look like. During the fitting procedure MoSeq repeatedly updates its view of what mouse behavior is in terms of the number, identity, and transition structure of syllables (through a statistical procedure called Gibbs sampling) to try to discover the best possible description of behavior. MoSeq also has another trick up its sleeve—it uses the hierarchical Dirichlet process, a Bayesian approach to statistical analysis, to cluster different instances of behavior into specific syllables. One key consequence of using this sort of probabilistic approach is that the number of behavioral syllables discovered by MoSeq sublinearly scales with the amount of data—if you feed MoSeq a little data, you get a handful of syllables, but if you feed it more data the description of behavior emitted by MoSeq becomes correspondingly more complex. This is, of course, exactly how ethologists characterize behavior—when they first look at an animal in the wild they divide behavior up into a small number of parts because they aren’t exactly sure what they are looking at or how repeatable their observations are, but as they get more experience looking at a given animal in a given context, their confidence grows, allowing them to both better distinguish and group behaviors into categories. But this also means that there is no fixed answer to the question “how many syllables are there?”: the answer depends on how much data you have.

## If MoSeq assigns every frame a single label, then it by definition has to pick a single timescale at which it thinks behavior is organized. What timescale does MoSeq focus upon?

Behavior is, of course, organized at many timescales simultaneously. As a consequence, when segmenting behavior into parts, a core problem one has to address is that of timescale—do I want to know whether an animal is asleep or awake, or do I want to understand all the moment-to-moment fidgets during waking and sleep? One of the key discoveries that led to the development of MoSeq is that 3D mouse behavior is naturally rhythmic at the sub-second timescale—if you just look by eye (or with math), the presence of fast temporal structure in 3D mouse imaging data is obvious (Fig. 1b). Because of this finding, we have focused our analysis at the sub-second timescale by tuning a parameter that roughly defines the timescale at which MoSeq searches for behavioral structure within a given experiment; this prior is very flexible, however, which allows MoSeq to identify syllables whose mean duration ranges from 100 ms to more than a second. This sub-second timescale is particularly interesting because it corresponds to known neural dynamics that are relevant to rodent behavior, such as the amount of time it takes for information to traverse a cortico-striato-thalamic loop.

## So what has MoSeq taught us about the brain and behavior?

In both published and unpublished work, MoSeq has proved to be a flexible and general method for objectively describing the overall microstructure of behavior, one which enables researchers to easily identify behavioral changes (reflected as changes in the expression of individual syllables or syllable sequences) that are induced as a consequence of experimental manipulations [7–10]. MoSeq can discover previously hidden phenotypes in mutant mice, can explain the trial-by-trial behavioral consequences of optogenetic manipulations, and can capture differences in pose dynamics elicited by changes in the physical or sensory environment. It can describe the effect of pharmacological agents on behavior, and can identify both side effects and on-target effects in mouse models of disease. Increasingly, MoSeq is being used to reveal behavioral patterns that are characteristic of activation of particular neural circuits.

The structure of the behavioral model instantiated by MoSeq explicitly corresponds to the foundational hypothesis of ethology, that spontaneous and naturalistic behaviors are flexibly composed by the brain from a set of stereotyped parts. The finding that MoSeq discovers an underlying modularity to behavior at the sub-second timescale therefore suggests that the brain contains neural correlates for behavioral syllables, grammar, or both. To test this hypothesis, we have recently rendered MoSeq compatible with tethered optical or electrical neural recordings [10]. These experiments demonstrate that the dorsolateral striatum (DLS) contains explicit moment-to-moment neural correlates for behavioral syllables and grammar; further, activity in the DLS is required for the appropriate execution of behavioral sequences composed of syllables, but not for the implementation of syllables themselves. For example, mice exposed to the fox odor TMT generate a dramatic set of fear-related behaviors, including spatial avoidance of the fox odor and freezing. These motivated, odor-driven behaviors are not a consequence of the mouse generating new syllables, but rather are caused by changes in behavioral grammar that create new sequences from the same set of parts that is normally used during locomotor exploration (Fig. 1d, e). However, mice with DLS lesions do not generate fear-related behaviors in response to TMT, suggesting that the DLS is required to assemble syllables into adaptive behavioral sequences. Together these data suggest that MoSeq will be generally useful for characterizing complex relationships between the brain and behavior.

## Can’t behavior be divided up in many ways, all of which are equally meaningful or useful?

This is one of the core challenges that has vexed ethology since its founding—how does one decide how to divvy up behavior into units to allow subsequent analysis? There is little agreement on how to go about this. MoSeq takes a generative modeling approach, meaning that it is ultimately optimizing predictions of future behavior. While the atomization of sub-second behavior provided by MoSeq is certainly an approximation, we have generated many lines of evidence consistent with the notion that mouse behavior is modular, and that MoSeq gives us at least some access to that modular structure. From a modeling perspective, we have run a type of control called a cross-likelihood analysis that demonstrates that MoSeq does not invent modules where none exist—if we feed MoSeq synthetic behavioral data that lacks modules, MoSeq fails to impose structure on the data [7]. In addition, we have run model comparisons that demonstrate that describing behavior as being modular enables better predictions of future behavior than models in which behavior is described as being continuous. From a neurobiological perspective, the fact that we can identify explicit neural correlates for both syllables and grammar—and that the dorsolateral striatum contains within it a neural rhythm that correlates with switching from one syllable to the next—is a powerful implicit validation of the segmentation of behavior provided by MoSeq; the fact that lesions of the DLS alter syllable sequences but not the contents of the behavioral syllables themselves is also consistent with MoSeq capturing neural-driven structure in behavioral data [10]. All this said, we remain open-minded about our own core hypothesis, and continue to empirically explore the idea that the brain organizes mouse behavior out of modules.

## What are some other methods for dividing up spontaneous behaviors into units?

In the context of spontaneous behaviors expressed by unrestrained flies and rodents, most prior attempts at identifying behavioral modules have focused on grouping together poses or pose dynamics in one way or another without explicit reference to how these potential behavioral motifs are sequenced over time. The general approach is to take behavioral data, embed the data in a low dimensional space so that it is easy to visualize and analyze, and then apply some sort of clustering or segmentation algorithm to break behavior into components. These methods tend to use under-the-hood parameterization to specify the number of behavioral components in a given dataset. One powerful and generalizable approach in this vein is called MotionMapper, which takes video data, performs a wavelet transformation (which includes certain assumptions about the balance between spatial and temporal frequencies), embeds those data using t-SNE, and then uses watershedding to identify peaks in the t-SNE plot that correspond to behavioral components [11]. This embedding-based approach has an important advantage over MoSeq insofar as it can capture behaviors that are organized at drastically different timescales simultaneously; however, this benefit comes at the cost (relative to MoSeq) of ignoring information about behavioral sequences that helps to identify boundaries between behavioral components.

## How might you objectively compare different methods for categorizing mouse behavior?

Trained generative models make quantitative predictions about the structure of held-out testing data; as a consequence, prediction accuracy can be used as a yardstick to compare the ability of different generative models to capture how behavior might be organized within a given dataset. We took advantage of this feature of generative models in creating MoSeq; for example, we found that modeling behavioral syllables as autoregressive processes that evolve over time was superior (in terms of predicting behavior) to simply clustering the data using Gaussian mixture models [7]. As mentioned above, we also found that describing behavior as being composed of modules that switch one to the other over time made better predictions than describing behavior as a continuous process without any switching or modularity.

The ability to make these sorts of objective comparisons is, in and of itself, an argument for using these kinds of generative models going forward. If you think you have a better idea for how to describe behavior, you can write it down in model form, train the model on behavioral data, and then objectively compare its predictive performance to alternative generative models. This being said, in creating MoSeq we did not seek to simply maximally predict behavior, but instead to balance predictive power with interpretability, as our goal was to discover modularity in behavior (and to compare a modular model for behavior with alternatives). For example, one could use deep networks (a type of generative model) to model mouse behavior; these models would almost certainly outperform MoSeq at making predictions of future behavior, but the internals of a trained deep network model will lack the interpretability of a similarly trained MoSeq model, which represents behavioral modules and grammar explicitly in the model structure itself. Recent work from our lab attempts to blend the advantages of neural networks with the explicit model structure of MoSeq, and we anticipate this will be a fruitful avenue for future research [8].

Significant challenges also remain in comparing the performance of generative models like MoSeq with the output of non-generative behavioral characterization techniques like MotionMapper. Whether MoSeq, MotionMapper, or any other behavioral characterization approach is better or worse than any other will be dependent on the specific goals of a given experiment, and the specific data being fed each algorithm. Our laboratory has generated large-scale behavioral benchmarking datasets and is actively exploring ways of using these datasets to allow apples-to-apples comparisons amongst generative and non-generative methods, which in turn should yield insight into the relative strengths and weaknesses of each approach. As technical advances on the hardware and software side inevitably evolve, it will become more and more pressing to articulate approaches for rationally choosing amongst the diverse array of methods for quantifying behavior.

## OK, MoSeq sounds great, but 3D videos of mice look kinda blurry. Isn’t a high-resolution 2D image where I can see everything better than the low-resolution 3D movies used by MoSeq?

Not necessarily, for two reasons. First, there is a ton of covariance in mouse imaging data. Take the tail, for example; while the tail can be quite informative about a mouse’s behavioral state (such as the direction of a turn), that information is often redundant with other changes in the mouse’s body (like the specific pattern of bending of the mouse’s back during a turn). We find that—from a predictive perspective—there isn’t a lot of benefit to including high spatial frequency information in our analysis (we get very similar answers with different cameras whose spatiotemporal resolutions differ), and therefore to keep the computing snappy we often smooth out those details. Second, on a pixel-for-pixel basis, the low spatial resolution 3D data can often tell you more about pose dynamics than a high spatial resolution 2D image; this is because mice aren’t worms or walking flies—they naturalistically explore the world through 3D movements of their bodies and so express pose dynamics in 3D. Indeed, if we perform principal components analysis over the pixel data, the first principal component represents movement in the z-axis. That said, we can easily imagine a world in which the current single 3D camera setup used as input for MoSeq is surpassed by a different imaging system or data type. The critical thing is to have an objective and quantitative framework for understanding what a given change on the input side gives you in terms of predictive or descriptive power; as mentioned above, we are actively building analytical frameworks that will allow us and others to explicitly measure the benefits (and costs) of changes to the dimensionality and spatiotemporal resolution of raw behavioral data of different types.

## MoSeq seems pretty complicated to actually implement in my lab if I don’t already know how to code. I want something like Noldus Ethovision, where I press a button and analyzed data emerge. Can you help with this?

Yes! We are working with engineers to build a push-button system for MoSeq that also folds in much of the functionality provided by more conventional systems. We are designing the new platform so that it will be simple to use for beginners, but also will allow experts access to all important model parameters. We plan to hold classes as well—with luck all of this will be available relatively soon—so stay tuned. It is our hope that the availability of such a system will enable many labs with diverse goals and interests to explore complex behavioral phenotypes, although a push-button MoSeq platform will still require careful thinking about the meaning of the output in the context of a particular experiment. There are significant differences among the new methods for behavioral analysis that are being developed by many groups, but one universal area of agreement is that behavioral data—which is fundamentally high-dimensional, dynamical, and organized at timescales of milliseconds—should be given the same analytical respect that has thus far been reserved for neural data. And this means that any real understanding of behavior and its relationship to neural activity will require the active participation of researchers, rather than reliance upon automated systems. In our lab getting the output of a MoSeq model marks the beginning of a deeper analysis of the data, rather than an end in and of itself.

## What are some limitations of MoSeq?

The current version of MoSeq acquires data using a single, low temporal (e.g., 30 Hz) resolution 3D camera to image individual mice exploring simple arenas in the absence of complex objects; the analytical engine underlying MoSeq, which assumes that behavioral syllables are linear trajectories through pose space, then spits out a description of behavior limited to a single timescale. In addition, MoSeq cannot currently normalize differences in pose dynamics that are the consequence of mice being different sizes, preventing comparisons between fat and skinny mutant mice, for example, or a study of the evolution of behavior across development. MoSeq also is entirely concerned with global pose dynamics—it fails to consider fast rhythms (e.g., whisking, breathing), and in its current form has no access to limb dynamics, whose organization may be sharply different from those for pose. These constraints come on top of the natural challenges of working with any unsupervised description of behavior, in which it is often difficult to understand how a change in a behavioral component or sequence induced by an experimental manipulation is “meaningful” in the context of the animal’s ethology. It is important to note that alternative unsupervised behavioral analysis methods address some of these MoSeq-specific issues—MotionMapper, for example, captures behavior at multiple timescales simultaneously, and can more easily accommodate animals of different sizes.

## What is the future of Moseq?

We think of MoSeq as an evolving framework, in which we can swap out different kinds of inputs and different sorts of generative models to capture overall structure in behavior. In other words, we view the current version of MoSeq as an important first step, rather than a final product, and we are actively working on addressing the limitations to MoSeq articulated above by playing with different types of data, pre-processing strategies, and modeling approaches. We hope that ultimately MoSeq will enable flexible modeling of behavioral data of all sorts (ranging from ultrasonic vocalizations to point-tracking data, from accelerometer traces to whisking data), at many hierarchical levels simultaneously, in multiple ethologically relevant contexts, and across species (not just mice and rats, but also primate models and humans); we are also optimistic that MoSeq as a framework will eventually allow inference of joint dependencies (between stimuli and behavior, between pairs or groups of animals, between brain and behavior). Preliminary data suggest that reaching some or all of these goals should be possible. There is lots of work ahead of us, and of course there is a whole field that has been recently created to address these kinds of issues for behavior in general. It has been really exciting to watch computational ethology slowly but surely become a reality; we look forward to a future in which the resolution with which we probe the brain is matched by the resolution at which we characterize behavior.

## References

[1] Meyer AF, Poort J, O’Keefe J, Sahani M, Linden JF (2018). A head-mounted camera system integrates detailed behavioral monitoring with multichannel electrophysiology in freely moving mice. Neuron..

[2] Kabra M, Robie AA, Rivera-Alba M, Branson S, Branson K (2013). JAABA: interactive machine learning for automatic annotation of animal behavior. Nat Methods.

[3] Ben-Shaul Y (2017). OptiMouse: a comprehensive open source program for reliable detection and analysis of mouse body and nose positions. BMC Biol.

[4] Machado AS, Darmohray DM, Fayad J, Marques HG, Carey MR (2015). A quantitative framework for whole-body coordination reveals specific deficits in freely walking ataxic mice. eLife..

[5] Pereira TD, Aldarondo DE, Willmore L, Kislin M, Wang SSH, Murthy M (2019). Fast animal pose estimation using deep neural networks. Nat Methods.

[6] Mathis A, Mamidanna P, Cury KM, Abe T, Murthy VN, Mathis MW (2018). DeepLabCut: markerless pose estimation of user-defined body parts with deep learning. Nat Neurosci.

[7] Wiltschko AB, Johnson MJ, Iurilli G, Peterson RE, Katon JM, Pashkovski SL (2015). Mapping sub-second structure in mouse behavior. Neuron..

[8] Johnson M, Duvenaud DK, Wiltschko A, Adams RP, Datta SR (2016). Composing graphical models with neural networks for structured representations and fast inference. Adv Neural Inf Proces.

[9] Pisanello F, Mandelbaum G, Pisanello M, Oldenburg IA, Sileo L, Markowitz JE (2017). Dynamic illumination of spatially restricted or large brain volumes via a single tapered optical fiber. Nat Neurosci.

[10] Markowitz JE, Gillis WF, Beron CC, Neufeld SQ, Robertson K, Bhagat ND (2018). The striatum organizes 3D behavior via moment-to-moment action selection. Cell..

[11] Berman GJ, Choi DM, Bialek W, Shaevitz JW (2014). Mapping the stereotyped behaviour of freely moving fruit flies. J R Soc Interface.

